# Clinical implications of traction bronchiectasis in IPF and fibrotic RA-ILD – a retrospective single-center cohort study

**DOI:** 10.1186/s12931-026-03497-6

**Published:** 2026-01-13

**Authors:** Jakob Raith, Jannik Ruwisch, Jonas C Schupp, Theresa Graalmann, Nora Drick, Marius M Hoeper, Antje Prasse, Jan Fuge, Felix C Ringshausen, Leonard Knegendorf, Jessica Rademacher, Sabine Dettmer, Benjamin Seeliger

**Affiliations:** 1 Department of Respiratory Medicine and Infectious Diseases, Hannover Medical School, Hannover, Germany; 2 Biomedical Research in End-Stage and Obstructive Lung Disease (BREATH), Hannover Medical School (MHH), German Center for Lung Research (DZL), Hannover, Germany; 3 Section of Pulmonary, Critical Care, and Sleep Medicine, Department of Medicine, Yale School of Medicine, New Haven, CT USA; 4 Department for Rheumatology and Immunology, Hannover Medical School, Hannover, Germany; 5 Junior Research Group for Translational Immunology, TWINCORE, Centre for Experimental and Clinical Infection Research, Helmholtz Centre for Infection Research and the Hannover Medical School, Hannover, Germany; 6 Clinic for Pneumology, University Hospital of Basel, Basel, Switzerland; 7 Institute for Medical Microbiology and Hospital Epidemiology, Hannover Medical School, Hannover, Germany; 8 Centre for Experimental and Clinical Infection Research, TWINCORE, Helmholtz Centre for Infection Research and the Hannover Medical School, Hannover, Germany; 9 Institute of Diagnostic and Interventional Radiology, Hannover Medical School, Hannover, Germany

**Keywords:** IPF, RA-ILD, Bronchiectasis, Fibrosis

## Abstract

**Background:**

Bronchiectasis is a common feature in idiopathic pulmonary fibrosis (IPF) and rheumatoid arthritis-associated interstitial lung disease (RA-ILD). While these so-called traction bronchiectasis are often considered a secondary phenomenon in fibrosing ILD, their prognostic significance and relationship to respiratory pathogen detection and outcomes remain unclear.

**Methods:**

We conducted a retrospective, single-center cohort study in IPF or fibrosing RA-ILD patients with available high-resolution computed tomography (HRCT) and lower-respiratory tract microbial samples between 2014 and 2024. Bronchiectasis was assessed using the bronchiectasis subscore of the Brody score; fibrosis was quantified by deep-learning–based automated HRCT analysis. Primary outcome was 5-year transplant-free survival; secondary outcomes included isolation of pathogens per CDC criteria, PFT trajectories, bronchiectasis-associated symptoms, and hospitalization. Statistical methods included Cox regression, linear mixed-effects modeling and correlation analysis.

**Results:**

267 IPF and 56 RA-ILD patients were included. Median modified Brody score was 11.5 (IQR 7–16; max possible range 0–72). Higher Brody scores strongly correlated with fibrotic extent (*R* = 0.6, *P* < 0.001). Higher scores had significantly lower baseline FVC and DLCO (*P* < 0.001), but no differences in PFT trajectories over time. In multivariable Cox regression, higher bronchiectasis scores were independently associated with mortality (HR 1.03 per point [95%CI 1.01–1.06], *P* = 0.003); fibrosis extent showed similar results (HR 1.02, CI 1.00–1.03, *P* = 0.017). Pathogens were found at a median of 3 months after baseline in 50.9% (IPF) and 46.4% (RA-ILD), without association with survival, symptoms or Brody scores. *Staphylococcus aureus* was most common (28.9%); *Pseudomonas aeruginosa* was rare (1.9%).

**Conclusion:**

In both IPF and RA-ILD, higher bronchiectasis scores were associated with fibrosis extent and mortality, but not classical clinical bronchiectasis features. This supports traction bronchiectasis as a marker of fibrotic remodeling rather than a distinct syndrome.

**Trial registration:**

Not applicable.

**Supplementary Information:**

The online version contains supplementary material available at 10.1186/s12931-026-03497-6.

## Introduction

Idiopathic pulmonary fibrosis (IPF) is a progressive fibrosing interstitial lung disease (ILD), historically, associated with high morbidity and mortality, with patients historically surviving three to five years [1]. It is histologically defined by usual interstitial pneumonia (UIP) and characterized on high-resolution CT (HRCT) by honeycombing, reticular fibrosis and traction bronchiectasis, summarized as radiological UIP [2]. Other systemic diseases such as connective tissue disease (CTD) associated ILDs may present as progressive pulmonary fibrosis (PPF) [3]. Within CTD, rheumatoid arthritis (RA) is most frequently associated with ILD (10–60%) [4], with a significant proportion of patients developing PPF [5, 6], associated with considerable mortality [7]. Regardless of progression, fibrosing ILD can be detected in HRCT of up to 60% of patients with RA [8]. Traction bronchiectasis is frequently observed in both, IPF and RA-ILD and serves as a confident radiological proof of established fibrosis [2, 8].

Bronchiectasis in fibrosing ILDs is considered a result of mechanical distortion caused by fibrotic tissue remodeling. The surrounding scar tissue retracts, distending the airways, and creating so called traction bronchiectasis with its characteristic radiographic appearance [9]. Traction bronchiectasis in the setting of pulmonary fibrosis has been traditionally excluded from the definition of bronchiectasis disease, which is clinically characterized by chronic cough, sputum production, and recurrent pulmonary exacerbations [10–14]. Bronchiectasis outside the context of pulmonary fibrosis is considered a chronic inflammatory airway disease perpetuated by predominantly neutrophilic inflammation, impaired mucociliary clearance, and consequently recurrent and persistent bacterial infection, and structural lung damage [10, 15].

Nevertheless, the unspecific symptoms of clinically relevant bronchiectasis are also frequently reported by patients with IPF and other fibrosing entities [16–18]. Therefore, it is disputable whether bronchiectasis in pulmonary fibrosis is solely a bystander phenomenon due to mechanical tissue traction or an additional bronchial disease with clinical and prognostic features of bronchiectasis.

The aim of this study was to address the clinical and prognostic role of bronchiectasis, including traction bronchiectasis, in the context of fibrosing ILD by quantifying bronchiectasis extent and severity and its association to clinical outcomes including lung function, symptom burden, respiratory pathogen detection and survival.

## Methods

### Patient cohorts and study setting

**Fig. 1:**
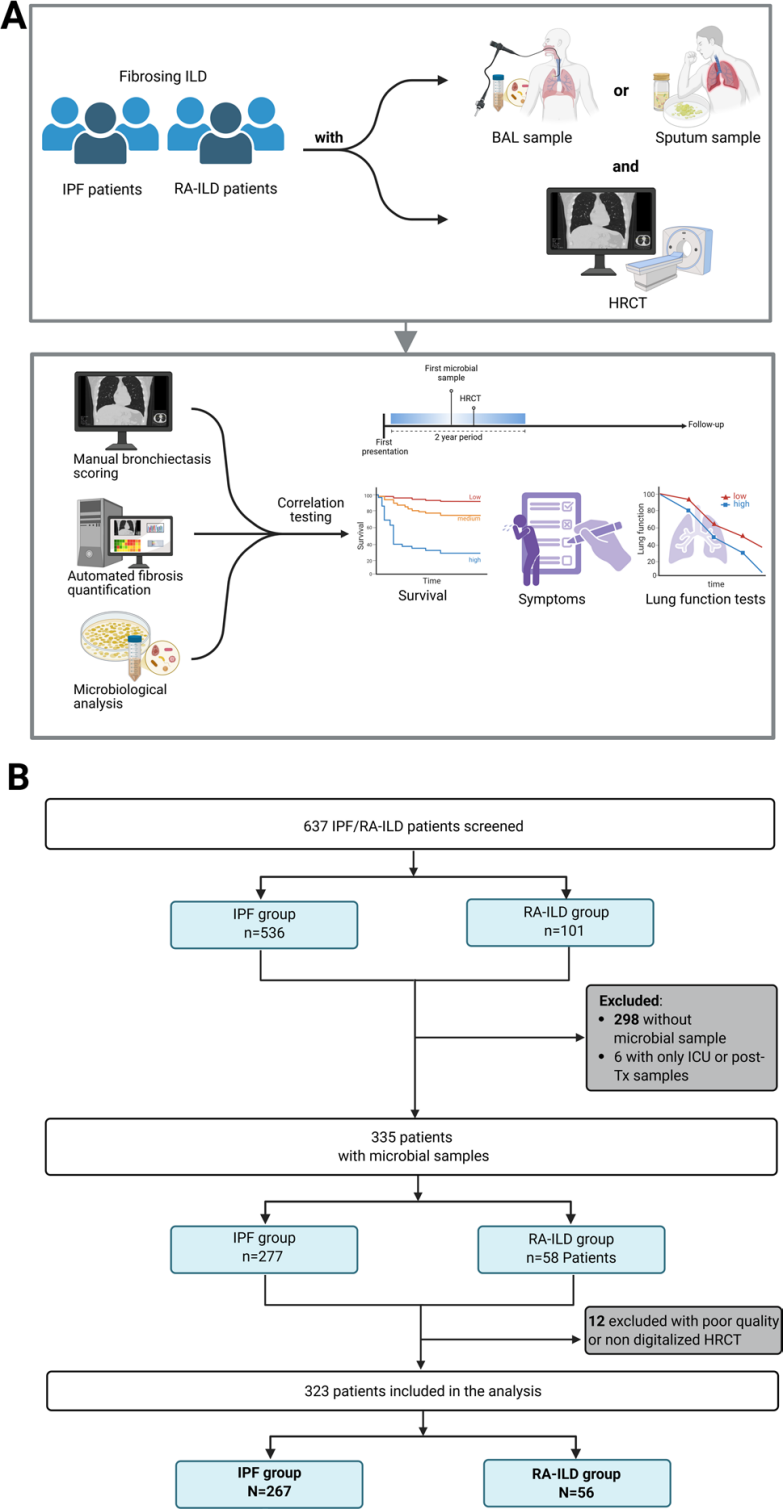
Study workflow. Schematic of study design integrating microbiological testing (bronchoalveolar lavage [BAL] and or sputum), high-resolution-computed tomography (HRCT) analysis, and correlation of bronchiectasis severity and fibrosis extent with survival, symptoms, and lung function (**A**). Flowchart of patient inclusion, resulting in 323 patients (IPF N = 267, RA-ILD N = 56) for final analysis (**B**)

In this study, we identified patients with a confident diagnosis of idiopathic pulmonary fibrosis (IPF) in accordance with the American Thoracic Society (ATS) and European Respiratory Society (ERS) guidelines [2] or rheumatoid arthritis-associated fibrosing interstitial lung disease (RA-ILD) as per ERS guidelines [19]. Patients were included in the analysis if they had a HRCT available for analysis and had lower-respiratory tract microbial samples via bronchoalveolar lavage (BAL) or sputum culture (Fig. 1A). All patients were evaluated and treated at a university hospital based specialized ILD clinic. The study was conducted in accordance with the 1964 Declaration of Helsinki and its later amendments. The study was approved by the institutional review board #11,931-BO-K-2025 and #2923 − 2015. All patients provided written informed consent.

Baseline and follow-up data were collected, including demographics, comorbidities, smoking status, pulmonary function tests (PFTs) and DLCO measurements as per ATS/ERS guidelines [20]. As symptoms, cough, night sweats, heartburn, sputum production, and weight loss were recorded. From February 2018 onward, use of anti-infectives were systematically recorded using a standardized questionnaire. PFT were performed at each clinical visit, which were scheduled according to guideline recommended intervals of 3 to 6 month. There were no predefined, fixed time points for PFT assessments across the cohort. All available measurements were analyzed. When BAL was performed as previously described [21], percentage of neutrophils was recorded. Additionally, the gender-age-physiology (GAP) score and index were calculated [22]. The GAP index is frequently used as a surrogate marker for disease severity [23]. Originally validated as a mortality prediction tool in IPF, it was similarly prognostic in RA-ILD [24].

### Computed tomography evaluation

#### Bronchiectasis scoring

Bronchiectasis severity and extent were assessed using these subdomains of the scoring system based on the method described by Brody et al. [25, 26], applied to each lung lobe, including the lingula as a separate lobe. For each patient, the HRCT scan closest to the first date of the microbiological sample was selected. All terms were used according to the Fleischner Society definition [27]. Bronchiectasis was diagnosed by the presence of one or more of the following criteria: a bronchoarterial ratio > 1, a non-tapering bronchus, a bronchus visible within 1 cm pleura surface [28]. In contrast to Brody, we did not assess mucus plugging, peribronchial thickening, parenchymal changes, or air trapping, but focused exclusively on the bronchiectasis subscore, thereby modifying the original protocol. Parenchymal changes as per Brody are confounded by IPF/RA-ILD changes, which were thus independently quantified as outlined below. Therefore, the extent of bronchiectasis was evaluated on a scale from 0 to 3 (0 = none; 1 = less than 1/3 of the lobe; 2 = 1/3–2/3 of the lobe; and 3 = more than 2/3 of the lobe) separately for the central and in the peripheral lung. The sum of both was multiplicated with the average multiplier size (0.5 = 0; 1 = 1; 1.5 = 1.25; 2.0 = 1.5; 2.5 = 1.75 and 3 = 2). The multiplier size was calculated by the sum of the largest dilated bronchus (1 ≤ 2x; 2 = 2x-3x; 3 ≥ 3x the size of the accompanying vessel) and the average size of dilated bronchi (1 ≤ 2x; 2 = 2x-3x; 3 ≥ 3x the size of the accompanying vessel) divided by 2. The score was applied to each lung lobe with the lingula as separate lobe resulting in a maximum possible score of 72 with higher scores indicating more severe bronchiectasis [25].

#### Quantification of fibrosis

HRCT scans were analyzed using a deep learning–based software tool (AVIEW Lung Texture, Coreline Soft, Seoul, Republic of Korea) to quantify fibrotic lung changes [29, 30]. This system automatically classifies the lung parenchyma into six patterns: normal lung, reticulation, honeycombing, ground-glass opacity, consolidation, and emphysema. Reticulation and honeycombing were identified as fibrotic patterns and the combined percentage of the total lung volume was calculated [31].

#### Microbiological evaluation

Sputum and BAL samples were collected during routine clinical evaluation and processed at the hospital’s ISO 15,189-accredited microbiology laboratory. BAL is performed in most patients as part of routine work-up of ILD. Sputum was collected in patients with increased sputum production, as clinically indicated. All relevant microbiological data were retrieved directly from the laboratory information system database using SQLAlchemy (version 2.0) in a Python (version 3.9) environment.

Microbiological assessment was conducted based on the CDC pneumonia criteria, which define potential respiratory pathogens based on a combination of clinical, radiological, and microbiological findings [32]. Specifically, sputum and BAL samples were analyzed for bacterial and fungal pathogens that met the CDC-defined criteria for pneumonia-associated organisms. No differentiation between colonization and sole isolation of pathogens was made due to unavailability of all clinical information at each time of the sampling. Thus, all pathogen isolation was regarded as “pathogen detection”.

#### Data analysis

All analyses were conducted using R (version 4.4.2, R Foundation for Statistical Computing, Vienna, Austria). Categorical variables were compared using chi-squared tests. Continuous variables were reported as medians with interquartile ranges (IQR) and analyzed using Kruskal–Wallis or Wilcoxon test, as appropriate. Statistical significance was defined as a two-sided p-value < 0.05.

To evaluate the association between bronchiectasis extent and extent of fibrotic changes, Spearman’s rank correlation coefficient was calculated. For visualization of subgroup analyses, patients were categorized into tertiles based on their total modified Brody score.

Longitudinal trajectories of PFTs were assessed by means of linear mixed-effects models adjusting for diagnosis, time and the modified Brody score as fixed effects. The whole model is shown in eTable1. FVC and DLCO were investigated as relative values to baseline. To assess the uncertainty in the fixed effects and the predicted longitudinal trajectories, we performed non-parametric bootstrapping with replacement to estimate empirical model related confidence intervals based on 1000 iterations. Visualization of the variation in iterated fixed effects estimates and the predicted trajectories (*lmerTest*) was performed with *ggplot2*.

Symptom profiles were compared across modified Brody score tertiles. For symptoms, patients without available data were excluded from the analysis.

Transplant-free survival was assessed using Kaplan–Meier estimates and log-rank testing. Multivariable Cox proportional-hazards models were used to determine the prognostic relevance of bronchiectasis severity vs. fibrotic extent. Models were adjusted for diagnosis, detection of respiratory pathogens, and GAP-points. Patients were censored in case of loss to follow-up.

## Results

### Patient cohorts

After exclusion of patients without available microbiological samples and suitable HRCT, 267 patients with IPF and 56 patients with RA-ILD were included in the final analysis (Fig. 1B). Within the RA-ILD group, 48 (85.7%) patients were rheumatoid factor positive and 42 (75%) had anti-CCP-antibodies. 16.5% were female in the IPF cohort vs. 51.8% in the RA-ILD cohort. The median age at the first presentation was similar with 69.5 years (IQR 61.9–74.9) in IPF vs. 66.9 years (IQR 59-72.7) years in the RA-ILD group (Table 1). Baseline PFTs were overall more preserved in the RA-ILD-group with a median FVC of 79% (IQR 62.5–90.5) compared to 68% of predicted (IQR 54–81) for IPF (*P* = 0.001). Comorbidity profiles were similar. In the RA-ILD group, 91.1% had received any immunosuppressive agents at least once during follow-up, vs. 43.1% in the IPF-group (*P* < 0.001). At the last available follow-up, 73.2% of the RA-ILD group, and 31.2% in the IPF-group received low dose systemic corticosteroid, 57.1% in RA-ILD and 3.8% in IPF received any other immunosuppressive agents (*P* < 0.001). 85% of IPF patients were treated with antifibrotic therapy during follow-up vs. 48.2% in the RA-ILD group (*P* < 0.001). A trial of nebulized hypertonic saline (6%) was used in 40.8% of IPF vs. 46.4% of RA-ILD patients during the follow-up period.

**Table 1:** Baseline demographics

Characteristics, *n* (%) or median (IQR)	All patients (*n* = 323)	IPF cohort (*n* = 267)	RA-ILD cohort (*n* = 56)	*P*-value (IPF vs. RA-ILD)
Age at first presentation, years	69.5 (61.9–74.9)	69.9 (62.8–75.1)	66.9 (59.7–72.7)	0.054
Female gender	73 (22.6)	44 (16.5)	29 (51.8)	< 0.001
Total follow up time, months	29.4 (11.2–50.0)	30.8 (11.1–50.5)	27.9 (12.8–45.6)	0.484
BMI, kg/m²	28.0 (25.4–31.3)	28.0 (25.6–31.3)	28.0 (23.9–31.1)	0.536
GAP Stage				
I	125 (38.7)	95 (35.6)	30 (53.6)	
II	132 (40.9)	112 (41.9)	20 (35.7)	
III	66 (20.4)	60 (22.5)	6 (10.7)	
Pattern				
UIP	-	-	34 (60.7)	
NSIP and other	-	-	22 (39.3)	
Serology				
Rheumatic Factor	-	-	48 (85.7)	
anti-CCP	-	-	42 (75)	
Comorbidities				
Diabetes mellitus	46 (14.2)	41 (15.4)	5 (8.9)	0.298
Arterial Hypertension	117 (36.2)	96 (36)	21 (37.5)	0.948
Coronary artery disease	87 (26.9)	75 (28.1)	12 (21.4)	0.392
COPD	37 (11.5)	28 (10.5)	9 (16.1)	0.336
Pulmonary hypertension	22 (6.8)	17 (6.4)	5 (8.9)	0.689
GERD	23 (7.1)	22 (8.2)	1 (1.8)	0.147
PFT at baseline (% predicted)				
Forced vital capacity	69 (54-83.2)	68 (54–81)	79 (62.5–90.5)	0.001
DLCO	54 (41–66)	53 (40–65)	61 (51–78)	0.002
FEV_1_/FVC (%)	86.2 (81.1–92.3)	86.3 (81.5–91.3)	84.9 (80–91)	0.620
Capillary blood gases (mmHg)				
pO_2_	70.9 (63.9–78.3)	70.2 (63.5–78.0)	74.3 (66.6–79.7)	0.833
pCO_2_	38.2 (35.8–40.7)	38.2 (35.8–40.8)	37.8 (35.8–40.6)	0.042
LTOT	38 (11.8)	35 (13.1)	3 (5.4)	0.115
Immunosupressive therapy during follow ups	166 (51.4)	115 (43.1)	51 (91.1)	< 0.001
cDMARD/Cyclophosphamide during follow ups	41 (12.7)	19 (7.1)	22 (29.7)	< 0.001
B cell-depleting biologicals during follow ups	23 (7.1)	2 (0.7)	21 (37.5)	< 0.001
Prednisolone (any dose) during follow ups	156 (48.3)	110 (41.2)	46 (82.1)	< 0.001
Immunosupressive therapy at last visit	138 (42.7)	88 (33.9)	50 (89.3)	< 0.001
cDMARD/Cyclophosphamide at last visit	28 (8.7)	9 (3.4)	19 (33.9)	< 0.001
B cell-depleting biologicals at last visit	20 (6.2)	1 (0.4)	19 (33.9)	< 0.001
Prednisolone (any dose) at last visit	126 (39)	85 (31.2)	41 (73.2)	< 0.001
Antifibrotic treatment (last visit)	25 (78.7)	227 (85)	27 (48.2)	< 0.001
Nintedanib	194 (59.9)	167 (62.5)	27 (48.2)	0.003
Pirfenidone	64 (19.8)	64 (24.0)	0	< 0.001

*IPF* Idiopathic pulmonary fibrosis, *RA-ILD *Rheumatoid arthritis-associated interstitial lung disease, *IQR *Interquartile range, *BMI *Body mass index, *GAP *Gender-age-physiology index, *UIP *Usual interstitial pneumonia, *NSIP *Nonspecific interstitial pneumonia, *anti-CCP *Anti-cyclic citrullinated peptide antibodies, *COPD *Chronic obstructive pulmonary disease, *GERD *Gastroesophageal reflux disease, *PFT *Pulmonary function test, *DLCO* Diffusing capacity of the lungs for carbon monoxide, *FEV*_1_Forced expiratory volume in one second, *FVC *Forced vital, *LTOT *Long-term oxygen therapy, *cDMARD* conventional disease-modifying antirheumatic drug

### Bronchiectasis score and lung fibrosis quantification

**Fig. 2:**
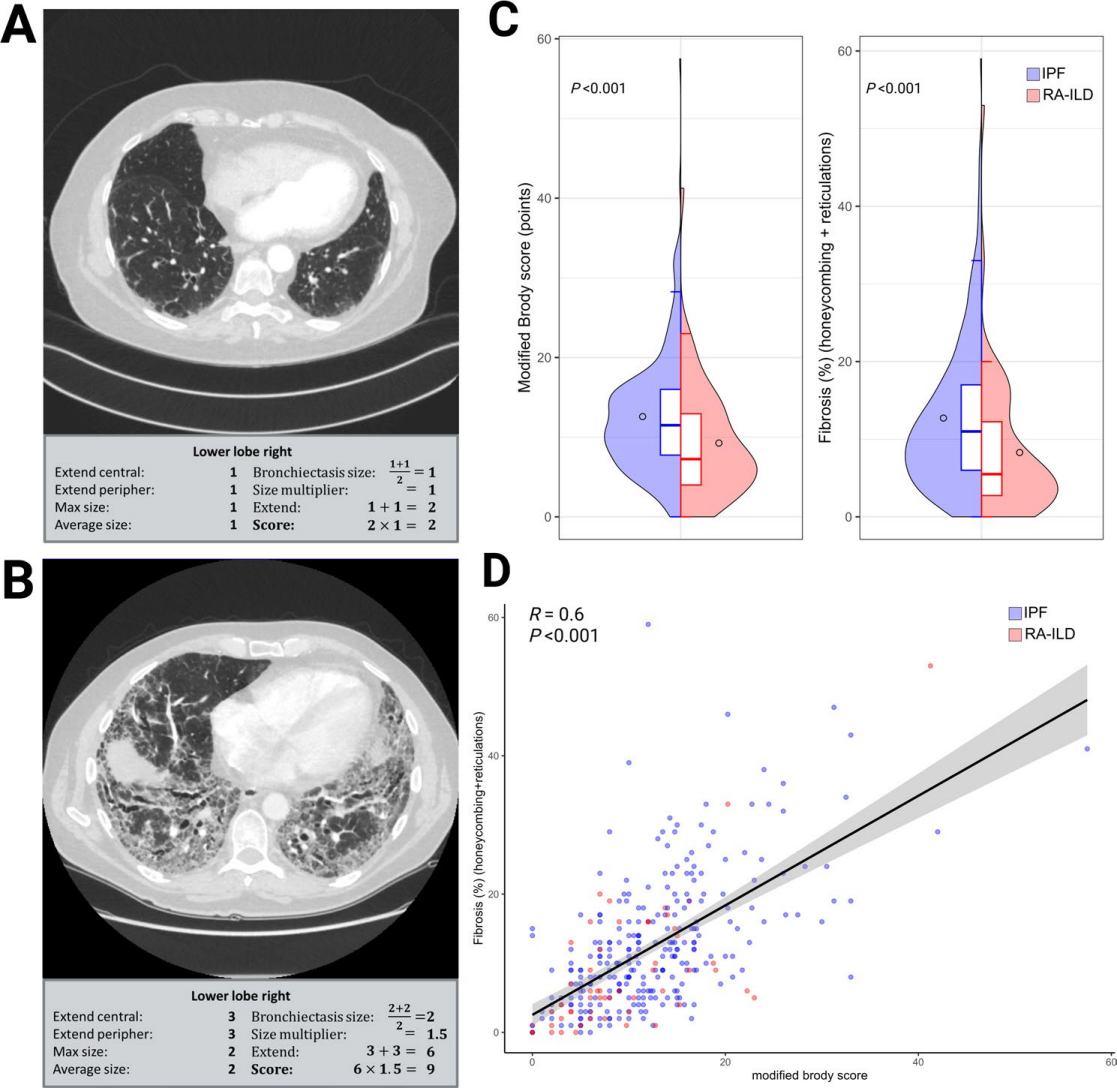
Radiological severity of bronchiectasis and correlation with fibrosis extent on HRCT. Representative HRCT image of the right lower lobe with a low modified Brody score of 2 (**A**). Representative HRCT image of the right lower lobe with a high modified Brody score of 9 (**B**). Distribution between diagnosis groups of modified Brody score and HRCT quantification of fibrosis using Wilcoxon test (**C**). Correlation between modified Brody score and quantified fibrosis using the Spearman’s rank correlation coefficient (**D**)

The modified Brody score was different between the groups with a median of 11.5 (IQR 7.9–16) in the IPF group and 7.2 (IQR 4-12.9) in the RA-ILD group (*P* < 0.001) (Fig. 2C). All subdomains, consisting of extent and severity components, showed similar results with higher scores in the IPF cohort (Table 2). Equally, total fibrosis extent quantified by HRCT was significantly higher in the IPF group (11% [IQR 6–17] vs. 5.5% [IQR 2.8–12.2], *P* < 0.001). A remarkably strong positive correlation between the bronchiectasis scores and the proportion of fibrotic HRCT changes as determined was evident with a Spearman correlation coefficient of *R* = 0.6, *P* < 0.001 (Fig. 2D).

**Table 2:** HRCT bronchiectasis scoring and fibrosis quantification with subdomains

Characteristics, median (IQR)	All patients (*n* = 323)	IPF cohort (*n* = 267)	RA-ILD cohort (*n* = 56)	*P*-value (IPF vs. RA-ILD)	Spearman *R* (bronchiectasis vs. fibrosis)	*P*-value (bronchiectasis vs. fibrose)
**Bronchiectasis score**	**11 (7-15.5)**	**11.5 (7.9–16)**	**7.2 (4-12.9)**	**< 0.001**	**0.6**	**< 0.001**
**Subdomains**						
Extent central	4 (2–6)	4 (3–6)	3 (2-5.2)	0.006	0.45	< 0.001
Extent peripheral	6 (4–8)	6 (5–8)	4.5 (2–7)	< 0.001	0.67	< 0.001
Max size	6 (4–7)	6 (4.5-7)	5 (2.9–6.5)	< 0.001	0.52	< 0.001
Average size	6 (4–6)	6 (4–6)	5 (2.8-6)	0.002	0.53	< 0.001
**Total fibrosis (%)**	**10 (5–16)**	**11 (6–17)**	**5.5 (2.8–12.2)**	**< 0.001**		
Honeycombing (%)	0 (0–2)	0 (0–2)	0 (0–1)	0.107		
Reticulations (%)	9 (5–14)	10 (6–15	5 (2.8–10)	< 0.001		

Data are shown as median (IQR). P-values refer to group comparisons (IPF vs. RA-ILD). Spearman R indicates correlation with fibrosis extent on HRCT

*IPF* Idiopathic pulmonary fibrosis, *RA-ILD *Rheumatoid arthritis-associated interstitial lung disease, *IQR *Interquartile range, *HRCT* High-resolution computed tomography

### Lung function trajectories

**Fig. 3:**
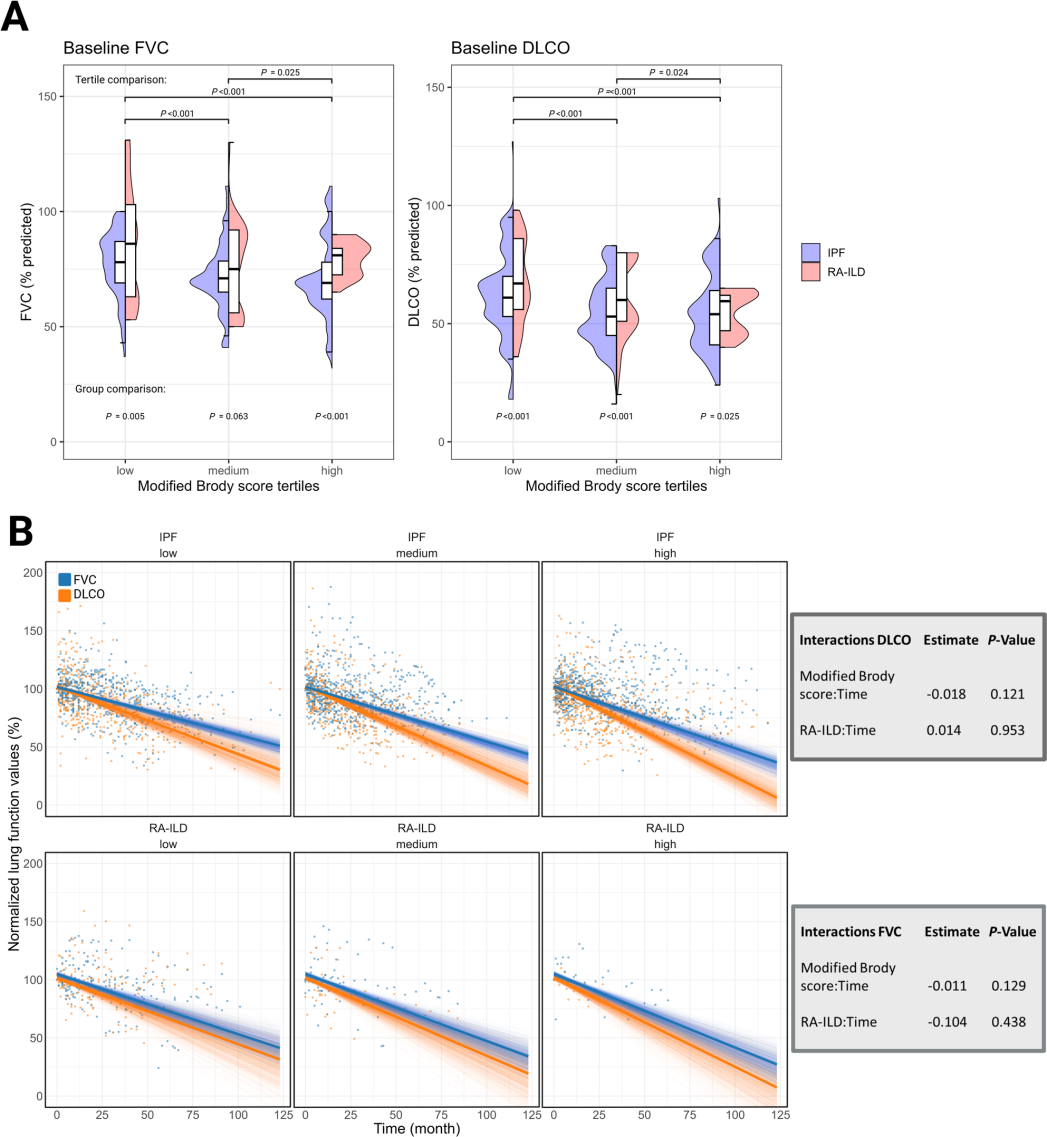
Association of bronchiectasis extent with baseline and longitudinal pulmonary function tests. Split violin and boxplots showing baseline PFTs across modified Brody score tertiles in IPF and RA-ILD patients. Comparisons used Wilcoxon test (**A**). Modeled longitudinal changes in PFTs over time by modified Brody score and diagnosis group. Solid lines represent fixed-effect predictions from linear mixed-effects models, transparent lines showing bootstrapped estimates across 1000 iterations. (**B**) There was no significant interaction between the Brody score and longitudinal FVC and DLCO trajectories

For further analyses, bronchiectasis scores were stratified into tertiles. Baseline values of FVC and DLCO, both expressed as percentages of predicted values, differed significantly across the tertiles, with higher bronchiectasis scores associated with lower baseline lung function (*P* < 0.001) (Fig. 3A). While baseline values differed, no significant differences in the trajectories of both FVC and DLCO were observed between modified Brody scores over time in both groups, respectively (Fig. 3B). In IPF, mean relative annualized FVC loss from baseline increased by 0.13% per modified Brody score point, corresponding to estimated losses of 5.1%, 5.6%, and 6.2% at scores of 7, 11, and 15.5, respectively. In RA-ILD, the respective declines were 6.3%, 6.9%, and 7.4% (eTable 1).

### Patient reported bronchiectasis-associated symptoms

**Fig. 4:**
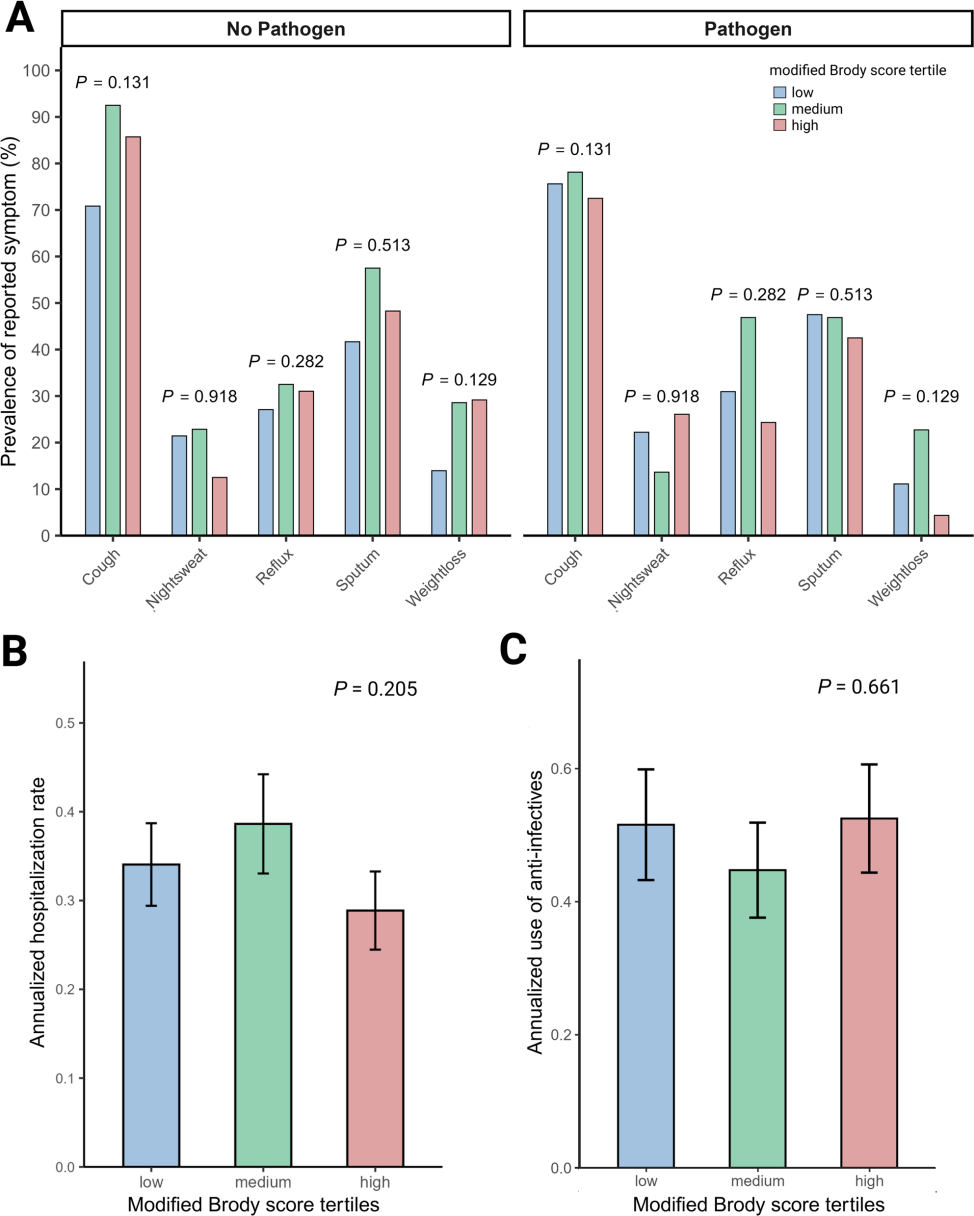
Prevalence of symptoms hospitalization rates and exacerbation rates across modified Brody score tertiles. Prevalence of key symptoms at first presentation stratified by pathogen detection status and modified Brody score tertiles. Comparison with Chi-squared test (**A**). Annualized hospitalization rates across modified Brody score tertiles with Kruskal-Wallis test (**B**). Annualized exacerbation rates across modified Brody score tertiles with Kruskal-Wallis test (**C**)

Overall bronchiectasis-related symptom burden was high in both groups: cough was reported by 79.1% of IPF and 73.2% of RA-ILD patients. Sputum (most days of the week) was reported in 48.1% of IPF and 42.9% of RA-ILD patients. No significant differences in the prevalence of these symptoms were observed between the bronchiectasis tertiles (Fig. 4A). The identification of a respiratory pathogen in any microbiological sample did not significantly affect symptom prevalence. Similarly, the annualized hospitalization rate (*P =* 0.205) and the annualized use of anti-infectives did not differ significantly across the tertiles (*P* = 0.661) (Fig. 4B, C).

### Survival

**Fig. 5:**
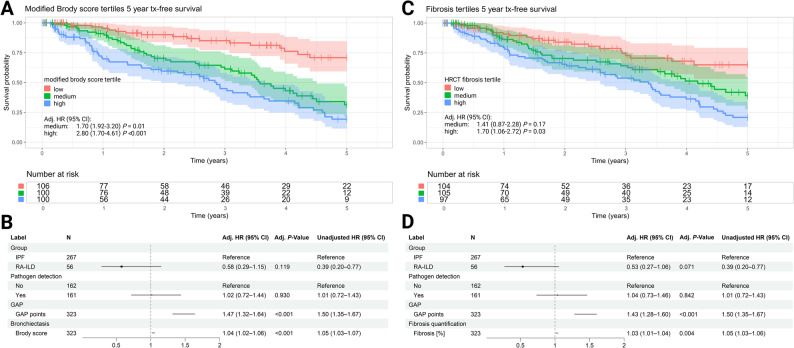
Impact of bronchiectasis and fibrosis extent on 5-year transplant-free survival. Kaplan-Meier curves for 5-year transplant-free survival by modified Brody score tertiles (**A**). Forest plot of adjusted Cox regression model for risk of 5 year transplant or death by total modified Brody score (**B**). Kaplan-Meier curves for 5-year transplant-free survival by HRCT fibrosis extent tertiles (**C**). Forest plot of adjusted Cox regression model for risk of 5 year transplant or death by fibrosis extent (**D**)

Survival analysis showed significant differences across bronchiectasis score tertiles, with lower 5-year transplant-free survival with increasing bronchiectasis tertile (low tertile: 70.8%, medium tertile: 31.9%; high tertile 19.3%) **(**Fig. 5A**)**. Over the observation period, a total of 21 IPF patients and 2 RA-ILD patients received lung-transplantation, 111 IPF and 7 RA-ILD patients died. In multivariable Cox regression models, the absolute bronchiectasis score remained an independent predictor of mortality (adjusted HR 1.04 [95%CI 1.02–1.06], *P* < 0.001) (Fig. 5B). Similar findings were observed for the extent of fibrotic lung involvement. Higher fibrosis tertiles were associated with reduced survival, and absolute fibrosis percentage also remained a significant predictor in the adjusted model (adjusted HR 1.03 [95% CI 1.01–1.04], *P =* 0.004) (Fig. 5C, D).

### Microbiological results

**Fig. 6:**
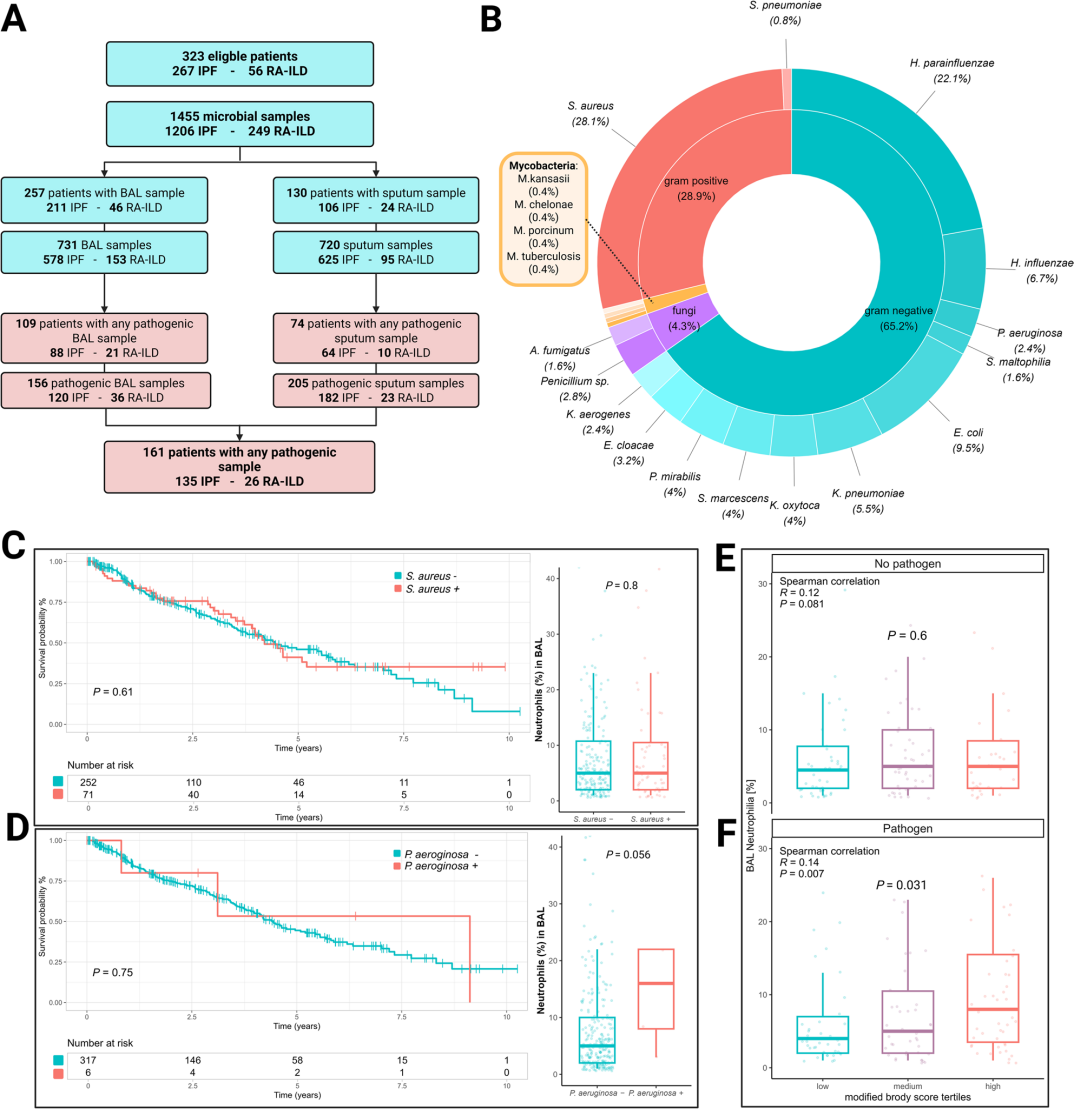
Spectrum of pathogen detection and BAL neutrophilia by bronchiectasis extent. Flowchart of microbiological sampling and detection of pathogenic organisms (**A**). Overview of detected pathogens and distribution (**B**). Kaplan-Meier survival curves with log-rank test and neutrophil percentages and Wilcoxon test in BAL with in patients with Staphylococcus aureus. (**C**) and Pseudomonas aeruginosa (**D**). BAL neutrophilia by modified Brody score tertiles in patients without pathogen detection compared via Kruskal-Wallis test (**E**). BAL neutrophilia by modified Brody score tertiles in patients with pathogen detection compared via Kruskal-Wallis test (**F**)

Microbiological sampling included a total of 1455 respiratory specimens, comprising 720 sputum and 731 BAL samples (Fig. 6A). In total, 130 patients had at least one sputum sample available, and 257 patients underwent BAL. The mean number of samples per patient was 4.5 (± 4.9) and the median time from first presentation to first microbial sample was 3 months (IQR 0.5–23.3). The median absolute interval between microbiological sampling and HRCT was 4.4 months (IQR 1.8–9.7) Among these, 161 patients (135 IPF, 26 RA-ILD) had at least one pathogenic organism detected in either sample type (49.8%).

The most frequently isolated pathogens were *Staphylococcus aureus* (27.4%) and *Haemophilus parainfluenzae* (21.6%). *Pseudomonas aeruginosa* was detected in six IPF patients, while non-tuberculous mycobacteria were found in three patients. In one IPF patient, *M. tuberculosis* was isolated (Fig. 6B). Detailed distributions of detected pathogens across diagnostic groups are provided in eTable 2. The pathogen detection spectrum was similar between BAL and sputum (eTable 3).

Overall, detection of pathogens was not associated with overall survival (Fig. 5B and D). For pathogens of particular interest, no significant difference in overall survival was observed in patients colonized with *S. aureus* (*P =* 0.17) or *P. aeruginosa* (*P =* 0.94) (Fig. 6C, D). Presence of *P. aeruginosa* showed a trend of being associated with BAL neutrophilia (16% [IQR 8–22] vs. 5% [IQR 2–10], *P =* 0.056), but was not significant considering all respiratory pathogens (5% [IQR 2-9.5] without pathogen vs. 5% [IQR 3–13]) with pathogen, *P =* 0.06). When stratifying by the detection of any pathogen, BAL neutrophilia was significantly associated with increasing Brody scores with pathogen detection, but not in the absence of pathogens (*R* = 0.12; *P =* 0.081) (Fig. 6, E, F) Furthermore, the prevalence of detection of pathogens did not significantly differ between bronchiectasis tertiles (*P =* 0.342) (Fig. 6F).

## Discussion

In this single center cohort study with IPF and fibrosing RA-ILD, higher radiological bronchiectasis scores were closely correlated with the radiological extent of fibrosis. Higher modified Brody scores were associated with baseline PFT, but not with the magnitude of PFT decline over time. Bronchiectasis had a similar impact on survival as the quantification of fibrotic changes. As to bronchiectasis specific features, bronchiectasis scores did neither correlate with symptom burden or use of anti-infectives nor with the frequency of detection of pathogens in sputum or BAL. These findings support the view that the pathophysiology of traction bronchiectasis substantially differs from bronchiectasis not associated with IPF/RA-ILD.

To our knowledge this is the first study to quantify the extent and severity of bronchiectasis in fibrotic ILD with two of the most prevalent entities (IPF and RA-ILD) with correlation to serial PFTs and the analysis of pathogenic microbial isolates from respiratory samples.

At first, we quantified bronchiectasis by using a subscore of the Brody score [25]. The Brody score is well-established for evaluation of CT-changes in patients with cystic fibrosis (CF) and it is used for evaluation of disease severity, disease progression and therapy response. The Brody score was originally validated in young people with CF [25, 26], where association with symptoms and PFT trajectories has been demonstrated [25]. Later it was validated in bronchiectasis due to primary ciliary dyskinesia and patients post COVID-19 [33, 34], where it showed correlation with PFT and chronic cough. Compared to other entities, in both IPF and RA-ILD the prognostic and functional implications of bronchiectasis have not been systemically studied. Herein, we quantify subdomains of bronchiectasis in an IPF and RA-ILD cohort using this established radiological score.

We then correlated the overall modified Brody score which was strongly correlated with the overall radiological extent of fibrosis. One study correlating HRCT scans did assign a bronchiectasis severity score from 0 to 3 and found a high correlation with histological fibroblast foci score (*R* = 0.5; *P* < 0.0001) [35]. When assessing correlation of the ordinal bronchiectasis scores within the IPF/UIP subgroup, only the fibroblast foci score, but not overall reticulations or honeycombing were significantly correlated, which differs from our results [35]. The closer correlation observed in our study is best explained by more granular quantification of both bronchiectasis scores and fibrosis extent. The association with the fibroblast foci score however underscores the presumed mechanistical driver of traction bronchiectasis by advanced extracellular matrix deposition.

We did observe a significant correlation of higher Brody scores with neutrophilic inflammation as one of the traditional hallmarks of clinically relevant bronchiectasis [15], Albeit a trend was seen, there was no significant elevation of BAL neutrophilia with pathogenic isolates in the respiratory tract, with an overall spectrum of isolates which is not representative for bronchiectasis [36]. Notably, pathogen isolation rates did not increase with a high bronchiectasis score, as would be expected in bronchiectasis [37]. About 20% of the patients had pathogens of low virulence (i.e. *Haemophilus parainfluenzae*), which when detected only rarely prompt therapy [38, 39]. Exclusion of *H. parainfluenzae* in the analysis had no influence on the results on the survival analysis, pathogen detection rate between Brody tertiles and BAL neutrophilia. Neutrophilic inflammation was numerically higher in patients with *P. aeruginosa*, but due to small number of isolates, this difference did not reach statistical significance detectable and remained far below the expected number in bronchiectasis disease, typically 70–90% of BAL cells [40]. Pathogenic isolates were also not associated with adverse outcomes or symptoms in our cohort, underscoring the different clinical phenotype of traction bronchiectasis vs. bronchiectasis not associated with fibrosing ILD, which typically presents with chronic cough, chronic sputum production, frequent exacerbations of two per year, hospitalization rates around 26% per year and persistent infection with organisms such as *Pseudomonas aeruginosa*, *Haemophilus influenzae*, *Enterobacteriaceae* and *Staphylococcus aureus* [41]. While we detected these pathogens too, detection rates where far lower (28% vs. 76%) [41]. Host-microbial interactions in altered and more abundant microbiome in IPF has been proposed as persistent stimuli for alveolar injury in IPF [42], but we could not pin this to particular isolates in IPF patients herein.

Importantly, bronchiectasis without fibrosing ILD is a well described phenomenon in RA with increased morbidity compared to RA without bronchiectasis or bronchiectasis of other entities [43, 44]. Genetic predisposition by MUC5B promoter variant predisposed to development of RA-ILD [45]. In RA and also certain connective tissue diseases such as Sjoegren’s Disease, bronchi and bronchiole can be directly involved, i.e. in the setting of follicular bronchiolitis [46] which is associated with seropositivity [47, 48]. The symptomatic and bacterial spectrum in isolated RA-associated bronchiectasis was similar to other bronchiectases [49, 50]. This type of bronchiectasis secondary to RA was not subject of the present study and patients with isolated bronchiectasis outside of fibrotic area were not included herein.

These study results are strengthened by a multimodal assessment of two cohorts with fibrotic ILD, which in detail characterizes clinical impact of extent and severity of bronchiectasis. The results are however subject to the retrospective design without standardized timing of microbial sampling and variable follow-up and thus merely show associations and inform future prospective studies. Although sputum was only collected with suggestive symptoms, the pathogen spectrum was similar between BAL and sputum. In some cases no confident differentiation between pathogen colonization and isolation without clinical relevance could be made, especially we could not test bronchiectasis consensus criteria for chronic infection [14], thus we only referred to “pathogen detection”. However, the main goal was to define the spectrum of identified pathogens. We acknowledge the relatively high rates of exposure to corticosteroids, primarily driven by their short-term use during initial diagnostic evaluation, before changes in practice recommendations and in the context of acute clinical worsening. Even though some cohorts and large registries report similar use of systemic corticosteroid [51–54], immunosuppression in IPF should be avoided outside of acute exacerbation [55]. The arguably high rate of nebulized hypertonic saline use in both groups reflects the considerable symptom burden in our cohort. Another important limitation is the use of different CT protocols. However, we minimized this bias by excluding CT with insufficient quality. The lack of systematically recorded bronchiectasis exacerbation data is a limiting factor of this study. Although annual antibiotic use was assessed after February 2018 as a proxy, only 85.1% of patients were assessed in a structured manner.

## Conclusions

In this single center cohort with two groups of fibrotic ILD (IPF and RA-ILD), high bronchiectasis (modified Brody) scores correlated closely with fibrotic changes on HRCT, had no implications of PFT trajectory and provided no benefit over fibrosis quantification in terms of prognostication. Bronchiectasis associated with fibrosing ILD differed substantially in terms of symptoms and pathogen isolation as would be expected from clinically relevant bronchiectasis. To confirm these findings and directly compare these two different entities of bronchiectasis, a prospective multicenter study is needed.

## Supplementary Information

Supplementary Material 1.

Supplementary Material 2.

## Data Availability

A minimal dataset supporting the key findings of this study is available as an supplementary file.The full datasets analysed during the current study are not publicly available due to data protection and privacy regulations, but are available from the corresponding author upon reasonable request.

## Abbreviations

ATS: American Thoracic Society
BAL: Bronchoalveolar Lavage
BMI: Body Mass Index
CDC: Centers for Disease Control and Prevention
CF: Cystic Fibrosis
CI: Confidence Interval
CTD: Connective Tissue Disease
DLCO: Diffusing Capacity of the Lung for Carbon Monoxide
ERS: European Respiratory Society
FEV1: Forced Expiratory Volume in 1 Second
FVC: Forced Vital Capacity
GAP: Gender-Age-Physiology
HR: Hazard Ratio
HRCT: High-Resolution Computed Tomography
ILD: Interstitial Lung Disease
IPF: Idiopathic Pulmonary Fibrosis
IQR: Interquartile Range
LTOT: Long-Term Oxygen Therapy
NSIP: Nonspecific Interstitial Pneumonia
PFT: Pulmonary Function Test
RA: Rheumatoid Arthritis
RA-ILD: Rheumatoid Arthritis-associated Interstitial Lung Disease
UIP: Usual Interstitial Pneumonia
