# Supplementary material for: Clinical implications of traction bronchiectasis in IPF and fibrotic RA-ILD – a retrospective single-center cohort study
Source: Respir Res. 2026 Jan 13;27:31. doi: 10.1186/s12931-026-03497-6 (PMC12849085; doi:10.1186/s12931-026-03497-6)
Supplement: Supplementary file 1 — Supplementary Material 1. [file 12931_2026_3497_MOESM1_ESM.docx]

**Supplementary table 1**: Fixed effects estimates for baseline relative FVC and DLCO decline by modified Brody score tertile and disease group

|  | **Estimate** | **Std.error** | ***P*-value** |
| --- | --- | --- | --- |
| **FVC** |  |  |  |
| *Intercept* | 101.566707 | 0.69 | 0.000 |
| Modified Brody score | 0.016974 | 0.05 | 0.718 |
| Time (month) | -0.346468 | 0.1 | <0.001 |
| RA-ILD (Reference: IPF) | 3.154079 | 0.98 | 0.001 |
| *Interaction*: modified Brody score - Time | -0.010905 | 0.01 | 0.129 |
| *Interaction*: Time - RA-ILD | -0.104137 | 0.13 | 0.438 |
| **DLCO** |  |  |  |
| *Intercept* | 101.960260 | 1.16 | 0.000 |
| Modified Brody score | -0.031058 | 0.08 | 0.698 |
| Time (month) | -0.472893 | 0.17 | 0.007 |
| RA-ILD (Reference: IPF) | -0.608767 | 1.77 | 0.731 |
| *Interaction*: modified Brody score - Time | -0.018466 | 0.01 | 0.121 |
| *Interaction* Time – RA-ILD | 0.014238 | 0.24 | 0.953 |
|  |  |  |  |

RA-ILD = Rheumatoid arthritis associated interstitial lung disease; DLCO = Diffusing capacity of the lungs for carbon monoxide; FVC = forced vital capacity; IPF = Idiopathic pulmonary fibrosis;

**Supplementary table 2**: All pathogenic samples from sputum or BAL

| **Pathogen, n (%)** | **All (n = 323)** | **IPF (n = 267)** | **RA-ILD (n = 56)** |
| --- | --- | --- | --- |
| No pathogenic microbial detection | 161 (49.8) | 131 (49.1) | 30 (53.6) |
| **Gram positive** |  |  |  |
| *Staphylococcus aureus* | 71 (22.0) | 57 (21.3) | 14 (25.0) |
| *Streptococcus pneumoniae* | 2 (0.6) | 1 (0.4) | 1 (1.8) |
| **Gram negative** |  |  |  |
| *Haemophilus parainfluenzae* | 56 (17.3) | 49 (18.4) | 7 (12.5) |
| *Haemophilus influenzae* | 17 (5.3) | 12 (4.5) | 5 (8.9) |
| *Stenotrophomonas maltophilia* | 4 (1.2) | 3 (1.1) | 1 (1.8) |
| *Pseudomonas aeruginosa* | 6 (1.9) | 6 (2.2) | 0 |
| *Escherichia coli* | 24 (7.4) | 20 (7.5) | 4 (7.1) |
| *Klebsiella pneumoniae* | 14 (4.3) | 11 (4.1) | 3 (5.4) |
| *Klebsiella oxytoca* | 10 (3.1) | 9 (3.4) | 1 (1.8) |
| *Proteus mirabilis* | 10 (3.1) | 7 (2.6) | 3 (5.4) |
| *Serratia marcescens* | 10 (3.1) | 8 (3.0) | 2 (3.6) |
| *Enterobacter cloacae* | 8 (2.5) | 7 (2.6) | 1 (1.8) |
| *Klebsiella aerogenes* | 6 (1.9) | 5 (1.9) | 1 (1.8) |
| **Fungi** |  |  |  |
| *Penicillium sp* | 7 (2.2) | 6 (2.2) | 1 (1.8) |
| *Aspergillus fumigatus* | 4 (1.2) | 2 (0.7) | 2 (3.6) |
| **Mycobacteria** |  |  |  |
| *Mycobacterium chelonae* | 1 (0.3) | 1 (0.4) | 0 |
| *Mycobacterium porcinum* | 1 (0.3) | 0 | 1 (1.8) |
| *Mycobacterium tuberculosis* | 1 (0.3) | 1 (0.4) | 0 |
| *Mycobacterium kansasii* | 1 (0.3) | 1 (0.4) | 0 |

RA-ILD = Rheumatoid arthritis associated interstitial lung disease; IPF = Idiopathic pulmonary fibrosis;

**Supplementary table 3**: Comparison of pathogenic spectrum of BAL and sputum samples

| **Pathogen, n (%)** | **All pathogens detected (n = 253)** | **All pathogens in BAL (n = 145)** | **All pathogens in sputum (n = 130)** |
| --- | --- | --- | --- |
| **Gram positive** |  |  |  |
| *Staphylococcus aureus* | 71 (45.5) | 44 (30.3) | 35 (26.9) |
| *Streptococcus pneumoniae* | 2 (1.2) | 2 (1.4) | 0 |
| **Gram negative** |  |  |  |
| *Haemophilus parainfluenzae* | 56 (35.9) | 31 (21.4) | 28 (21.5) |
| *Haemophilus influenzae* | 17 (10.9) | 10 (6.9) | 7 (5.4) |
| *Stenotrophomonas maltophilia* | 4 (2.6) | 0 | 4 (3.1) |
| *Pseudomonas aeruginosa* | 6 (3.8) | 3 (2.1) | 4 (3.1) |
| *Escherichia coli* | 24 (15.4) | 14 (9.7) | 13 (10.0) |
| *Klebsiella pneumoniae* | 14 (8.9) | 8 (5.5) | 7 (5.4) |
| *Klebsiella oxytoca* | 10 (6.4) | 6 (4.1) | 4 (3.1) |
| *Proteus mirabilis* | 10 (6.4) | 8 (5.5) | 2 (1.5) |
| *Serratia marcescens* | 10 (6.4) | 4 (2.8) | 6 (4.6) |
| *Enterobacter cloacae* | 8 (5.1) | 4 (2.8) | 5 (3.8) |
| *Klebsiella aerogenes* | 6 (3.8) | 3 (2.1) | 5 (3.8) |
| **Fungi** |  |  |  |
| *Penicillium sp* | 7 (4.5) | 5 (3.4) | 2 (1.5) |
| *Aspergillus fumigatus* | 4 (2.6) | 3 (2.1) | 4 (3.1) |
| **Mycobacteria** |  |  |  |
| *Mycobacterium chelonae* | 1 (0.6) | 0 | 1 (0.8) |
| *Mycobacterium porcinum* | 1 (0.6) | 0 | 1 (0.8) |
| *Mycobacterium tuberculosis* | 1 (0.6) | 0 | 1 (0.8) |
| *Mycobacterium kansasii* | 1 (0.6) | 0 | 1 (0.8) |

RA-ILD = Rheumatoid arthritis associated interstitial lung disease; IPF = Idiopathic pulmonary fibrosis;
